# Beyond deployments: Australia’s strategic contributions to the Global Outbreak Alert and Response Network

**DOI:** 10.5365/wpsar.2024.15.5.1089

**Published:** 2024-06-13

**Authors:** Sharon Salmon, Kieh Christopherson, Stephanie Williams

**Affiliations:** a Indo-Pacific Centre for Health Security, Department of Foreign Affairs and Trade, Canberra, Australia.; b UNSW Medicine and Health, School of Population Health, University of New South Wales, Sydney, Australia.

The Global Outbreak Alert and Response Network (GOARN) was established by the World Health Organization (WHO) in April 2000 as a mechanism for technical partners and networks around the world to coordinate and assist WHO Member States in responding to public health emergencies. There are more than 300 GOARN partners, including technical institutions, organizations and networks, each willing to pool their resources to improve communication and information-sharing about emerging and ongoing public health events, and to support the capacities required for preparedness and rapid response activities. (*1*)

GOARN’s partners are national, regional and global stakeholders. Partners include ministries of health, national public health institutes, United Nations organizations, nongovernmental organizations, academic institutions, and surveillance, laboratory and technical networks. Each partner varies in its capacity, technical specialty, resources and ability to provide support.

Australia’s Indo-Pacific Centre for Health Security (CHS) is a branch of the Global Health Division of the Australian Government’s Department of Foreign Affairs and Trade. CHS, as part of Australia’s development assistance programme, provides institutional support to GOARN in ways that may serve as examples for other existing or potential partners to the Network.

CHS was established to enhance health security and strengthen public health responses in the Indo-Pacific region. CHS directs Australian aid funds to projects aimed at improving health systems and health security, sharing Australian expertise, and improving coordination to enhance the health and well-being of people and communities within the Indo-Pacific region.

Health emergency workforce development, including rapid outbreak response capability, has been a priority area for Australia’s health security investments through CHS since its establishment. (*2*) CHS joined GOARN as a global technical partner in health emergency response and coordination in 2018, and funded secondments to the WHO regional offices for South-East Asia and the Western Pacific the following year. Both CHS-funded roles are designated as GOARN technical officer positions. These positions were introduced to concentrate efforts on goals shared by CHS and GOARN: to increase the number of GOARN’s partners in both WHO regions, increase the engagement of existing South-East Asian and Pacific partners, and contribute to the development of the GOARN Strategy 2022–2026. (*3*)

The establishment of an Australian-funded position in the WHO Regional Office for the Western Pacific has led to measurable benefits for GOARN in the Region. Since February 2020, the Regional Office’s GOARN technical officer (SS) has facilitated 21 new partners to join the Network, including nine from Australia (**Box 1**). During the COVID-19 pandemic, close coordination between the GOARN technical officer in the Regional Office, WHO headquarters and country offices enabled the Network to navigate country entry requirements and travel restrictions for those being deployed by the Network, and ensured it was able to meet a diverse range of requests for assistance from WHO Member States. Between January 2020 and May 2023, 72 individuals completed 89 deployments to 12 Member States in the WHO Western Pacific Region. Of those deployed, 47% (34/72) were female. Thirty-four GOARN partner institutions provided technical experts for deployment, 16 of which were based within the Region. (*4*)

Box 1 **List of new Global Outbreak Alert and Response Network (GOARN) partners in the WHO Western Pacific Region since 2020**

**Table:** 

**Australia**
Asia Pacific Consortium of Veterinary Epidemiology
Clinical Excellence Commission, New South Wales Government
Collaborative for the Advancement of Infection Prevention and Control
National Critical Care and Trauma Response Centre
Queensland Infection Prevention and Control Unit, Queensland Health
School of Population Health, Medicine and Health, University of New South Wales
University of Newcastle
University of Western Australia
Sydney Infectious Diseases Institute, University of Sydney
**Cook Islands**
Public Health Directorate of the Ministry of Health, Te Marae Ora
**Guam**
Guam Department of Public Health and Social Services
**Japan**
Toshima Hospital
**Papua New Guinea**
Field Epidemiology Training Programme
**Philippines**
Association of Asia-Pacific Operational Research Societies
**Republic of Korea**
National Medical Center
**Singapore**
National Centre for Infectious Diseases
**Vanuatu**
Surveillance, Research and Emergency Response Unit, Ministry of Health
**Viet Nam**
Cho Ray Hospital
Pasteur Institute Ho Chi Minh City
Pasteur Institute Nha Trang
Tay Nguyen Institute of Hygiene and Epidemiology

In response to restrictive entry requirements across the Region, the technical officer supported the shift of the GOARN Tier 1.5 training – orientation to international outbreak response – from an in-person to a virtual format. Since February 2020, 380 individuals have completed this training, and 34 have been trained as trainers.

In addition to funding GOARN-focused positions in WHO regional offices, CHS has pursued other avenues to support GOARN’s work. As the COVID-19 pandemic evolved into a protracted emergency, Member States’ requests for assistance were for longer durations than GOARN’s usual 4- to 8-week deployments. CHS used a flexible mechanism to support income supplementation for longer deployments of Australian experts who would otherwise have been unable to respond to requests for assistance in the Region. CHS’s use of this mechanism was unique among GOARN’s institutional partners and, in certain circumstances, could continue to add value to the Network when available and appropriate.

CHS also seeks to make a positive contribution to GOARN’s governance and strategic direction. In 2022, a representative from CHS was elected as one of 21 partners to join the GOARN Steering Committee. The 4-year term provides CHS with the opportunity to further contribute to guiding the planning, implementation and evaluation of GOARN’s activities and strategic goals.

CHS intends to continue bolstering outbreak response capacity in the Indo-Pacific region through a range of mechanisms. This will include acting as an engaged institutional partner to support GOARN, as well as implementing Australia’s commitment to the Quad Health Security Partnership (*5*) to support outbreak preparedness and response training for the public health emergency workforce in South-East Asia. Each GOARN partner has the opportunity to support both their own and GOARN’s strategic goals through participation in the Network.
